# First report of an accident caused by *Jaguajir agamemnon* (C.L. Koch, 1839) (Scorpiones, Buthidae) in Paraná state, Brazil

**DOI:** 10.1590/0037-8682-0286-2023

**Published:** 2023-10-30

**Authors:** Fábio Henrique Kwasniewski, Emanuel Marques da Silva, Edmarlon Girotto, Denise Maria Candido, Miriam de Cássia Tóffolo, Daniel Emilio Dalledone Siqueira, Luiz Roberto Costa Gomes, Camilo Molino Guidoni

**Affiliations:** 1 Universidade Estadual de Londrina, Centro de Ciências Biológicas, Departamento de Ciências Patológicas, Londrina, PR, Brasil.; 2 Secretaria de Estado da Saúde do Paraná, Laboratório de Taxonomia de Animais Peçonhentos, Curitiba, PR, Brasil.; 3 Universidade Estadual de Londrina, Centro de Ciências da Saúde, Departamento de Ciências Farmacêuticas, Londrina, PR, Brasil.; 4 Instituto Butantan, Biotério de Artrópodes, São Paulo, SP, Brasil.; 5 Universidade Estadual de Londrina, Hospital Universitário, Centro de Informação e Assistência Toxicológica, Londrina, PR, Brasil.; 6 Secretaria de Estado da Saúde do Paraná, Centro de Informação e Assistência Toxicológica, Curitiba, PR, Brasil.; 7 Prefeitura Municipal de Entre Rios do Oeste, Entre Rios do Oeste, PR, Brasil.

**Keywords:** Scorpion stings, Jaguajir Agamemnon, Child

## Abstract

We report the first pediatric case of a *Jaguajir agamemnon* scorpion sting. This case occurred in the state of Paraná and is the first record of this species in southern Brazil. The patient was admitted within 15 min, with pain, local edema, erythema, whole-body pruritus, and tongue paresthesia within the first two hours, which disappeared thereafter. The patient’s condition was considered mild, with a positive outcome, and she recovered completely.

## INTRODUCTION

Scorpions are venomous animals that cause numerous human envenomations, especially in the poorest countries. Medically important stings are mainly caused by scorpions belonging to the Buthidae family, particularly those in the genus *Tityus* in South America^1^. In Brazil, mild accidents are typically characterized with local clinical manifestations, including pain, erythema, and edema, and sometimes with signs related to pain, such as mild tachycardia, nausea, and agitation. In moderate accidents, the systemic manifestations include sweating, nausea, vomiting, tachypnea, agitation, tachycardia, and hypertension. Severe envenomation is reported when such manifestations are intense, and others may be present, such as excessive salivation and sweetening, tachydyspnea, cardiac arrhythmias, hyper- or hypotension, agitation and prostration, convulsions, and muscle spasms, with progression to cardiogenic and respiratory shock causing death^2^.

Recently, scorpions have been involved in several accidents involving venomous animals in Brazil, posing an important public health issue. Most recorded accidents were caused by *T. serrulatus*, *T. bahiensis,* and *T. stigmurus*, which have similar clinical characteristics. When compared to the victims of any *Tityus*, who are generally implicated in mild accidents, people stung by *T. serrulatus* are at a higher risk of being included in moderate and severe accidents, particularly children aged 0-9 years, where lethality is also higher^3^.

Accidents involving different species of *Tityus* have been reported, especially in the northern area of the country, with symptoms distinct from those cited earlier^4^ ^-^ ^6^. However, stings have not been reported in other Buthidae scorpions. The new genus *Jaguajir* was created, and the species *Rhopalurus agamemnon* (C.L. Koch, 1839), *Rhopalurus pintoi* (Mello-Leitão, 1932), and *Rhopalurus rochae* (Borelli, 1910) were included as *Jaguajir agamemnon* (C.L. Koch, 1839), *Jaguajir pintoi* (Mello-Leitão, 1932), and *Jaguajir rochae* (Borelli, 1910), respectively^7^. They are commonly found in the north-eastern, mid-western, and northern regions of the Brazilian Cerrado.

Case descriptions of envenomation caused by *Jaguajir agamemnon* are extremely rare, and searches in the most used databases (Cochrane Library, LILACS, SciELO, Scopus, Redalyc, MedLine, and PubMed Central) showed no more than two articles describing accidents in adults living in the natural habitat of this scorpion. However, in only one case, the animal was properly identified^8^ ^,^ ^9^. Here, we report a pediatric case of *J. agamemnon*-induced envenomation in a small city in Paraná, southern Brazil.

## CASE REPORT

The accident occurred in February 2023 during the summer, involving a 6-year-old female child who was stung on the dorsum of the right foot while playing in her home in the urban area of the small city of Entre Rios do Oeste (24º42’14’’S, 54º14’32’’O), state of Paraná, in the South region of Brazil. The case was monitored via teleservice by the Center for Information and Toxicological Assistance of Londrina at the University Hospital of the State University of Londrina (CIATox-Londrina), a regional reference health service that provides 24-hour phone support or bedside consultations for toxicology and accidents, along with venomous animal clinical case management.

**FIGURE 1:**
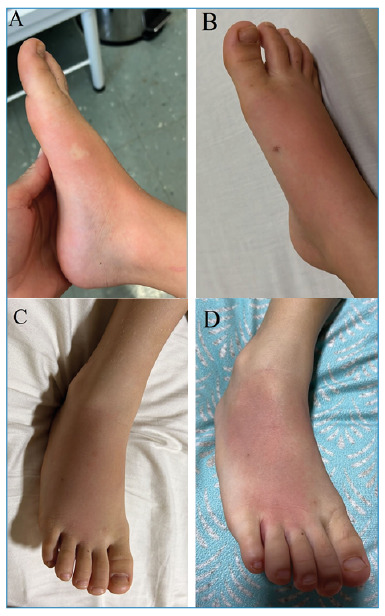
Images of the sting showing local edema and erythema **(A)**, a small area of hematoma in the local area of the sting and large erythema on the dorsum **(B)**, a swollen foot 24 h after the accident **(C)**, and persistent erythema on the dorsum of the foot 3 days later **(D)**.

The patient received the first medical care within 15 minutes in a Basic Health Unit (BHU) with intense pain and local signs of a sting surrounded by edema and erythema (Figure 1A). A local anesthetic blockade was performed, and the patient was referred to a medical clinic when contact with CIATox-Londrina was established. At this point, 30 min had elapsed since the sting. Analgesics were not administered, as the patient did not complain of any pain. Cleaning the injured site and verifying the vaccination card for tetanus is advised. In addition, the patient should be observed for at least 6 h with special attention to clinical manifestations such as sweating, agitation, tachycardia, hypertension, nausea, vomiting, and salivation. Approximately one hour after the sting, a small hematoma was observed at the sting site (Figure 1B).

Whole-body pruritus and tongue paresthesia resolved spontaneously two hours after the incident report, after which no further symptoms were observed. Between 2 and 4 h after the sting, the patient presented with stable health, with a mean arterial pressure of 90/60 mmHg, heart rate of 112-120 beats per minute, and oxygen saturation of 99%. The patient was continuously observed every 30 min before being discharged seven hours after she was admitted to the clinic. The patient’s condition was classified as mild and recovered fully. One day after the accident, the foot was swollen and erythematous (Figure 1C), and cold water compression was applied to relieve the edema. On the third day, erythema (Figure 1D) remained, and persistent tingling was observed in the toes.

**FIGURE 2:**
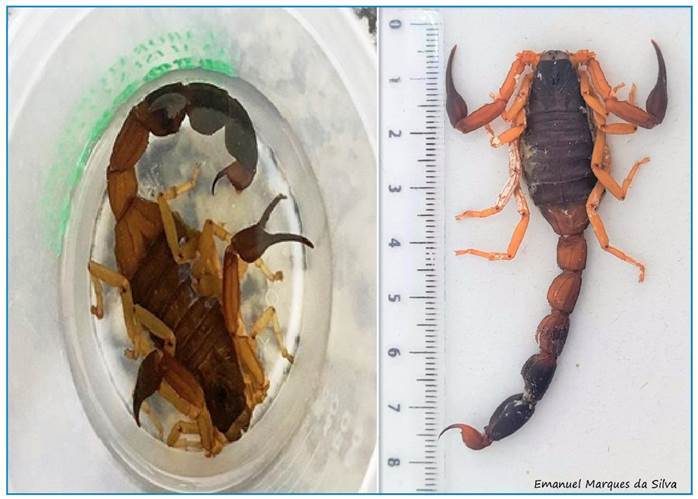
Images of the scorpion involved in the accident, identified as *Jaguajir agamemnon*.

The scorpion was captured by the family and sent to a municipal environmental health surveillance facility, which then forwarded its image for identification to the Venomous Animals Taxonomy Laboratory of the Center of Information and Toxicological Assistance of Paraná. It was identified as *Jaguajir agamemnon* (Figure 2).

## DISCUSSION

We are not aware of any pediatric case reports caused by *J. agamemnon*, making this the first report. In the State of Paraná, *T. bahiensis*, *T. serrulatus*, *T. costatus*, *Bothriurus,* and *Ananteris* spp. are frequently found. The State Program for the Surveillance of Accidents by Venomous and Poisonous Animals recorded two specimens of *J. agamemnon* in the 1990s from municipalities in the West that were never found again apart from sporadic cases of passive transport. Interestingly, the patient's father was a truck driver who had recently traveled to the northeastern region of the country. One study reported the occurrence of *J. rochae*, an animal adapted to the same biome as *J. agamemnon*, in the city of São Paulo (state of São Paulo, Southeastern Brazil), possibly arriving in boxes of melon^10^.

Although this case occurred in a child, it has some similarities with a previously published case regarding an adult who was stung by *J. agamemnon* in the state of Bahia, in the northeastern region of Brazil. Mild pain with intense local pruritus was reported, and after one hour, whole-body paresthesia with greater intensity was observed in the tongue and extremities^9^. Pruritus was present in our case but in the whole body, as well as paresthesia on the tongue. In our case, pain, local edema, and erythema were present soon after the sting; however, the edema and erythema persisted for up to three days. Although CIATox-Londrina pain was not reported at the time of follow-up, local anesthetic blockade was initially administered during the first visit to the BHU. One of the reasons why this type of approach to relieving local pain is no longer indicated is that it has a short-lived effect, and because the use of analgesics is no longer necessary, the pain is probably less intense.

This case and the one reported in Bahia^9^ were considered mild, while accidents involving an 18-year-old man and an adult woman caused by *J. agamemnon* in Piauí, northeast region, were considered moderate. In both cases, the local pain was intense, spreading to the shoulder in the young man; signs of systemic effects such as agitation, salivation, lacrimation, blurred vision, muscle spasms, tachycardia, and hypotension in the woman; and somnolence and salivation in the young man. Anti-scorpionic serum was prescribed in both cases. The scorpion was not captured at the time of these accidents; however, in the case of the female, it was captured afterward during a search of the surroundings of her property. In a case involving a young man, the animal was only described by the victim^8^.

Two more cases are documented in specialized literature with *Rhopalurus*, both in male adults living in the state and in the northeastern region of Brazil. In cases where the victim was stung by *R. amazonicus* (currently *R. laticauda* ^7^), local pain spread to the arm and paresthesia was felt^11^. In the case involving *J. rochae*, after starting with mild pain, the patient developed fatal anaphylaxis, as evidenced by autopsy findings of the glottis and pulmonary edema, and based on his history of allergy triggered by a bee sting and contact with beetles and crabs^12^.

In conclusion, cases involving *J. agamemnon* have rarely been reported. In addition to being described as the only pediatric case caused by this scorpion, this is the first reported case outside the northeast region. Although the outcome in our case was positive, more attention should be paid when the victim is a child, as moderate envenomation in adults has been previously reported.
